# Midgut pain due to an intussuscepting terminal ileal lipoma: a case report

**DOI:** 10.1186/1752-1947-4-51

**Published:** 2010-02-11

**Authors:** Noormuhammad O Abbasakoor, Dara O Kavanagh, Diarmaid C Moran, Barbara Ryan, Paul C Neary

**Affiliations:** 1 Division of Colorectal Surgery, Adelaide and Meath Incorporating the National Children's Hospital, Tallaght, Dublin 24, Ireland; 2 Department of Gastroenterology, Adelaide and Meath Incorporating the National Children's Hospital, Tallaght, Dublin 24, Ireland

## Abstract

**Introduction:**

The occurrence of intussusception in adults is rare. The condition is found in 1 in 1300 abdominal operations and 1 in 100 patients operated for intestinal obstruction. The child to adult ratio is 20:1.

**Case presentation:**

A 52-year-old Irish Caucasian woman was investigated for a 3-month history of intermittent episodes of colicky midgut pain and associated constipation. Ileocolonoscopy revealed a pedunculated lesion in the terminal ileum prolapsing into the caecum. Computed tomography confirmed a smooth-walled, nonobstructing, low density intramural lesion in the terminal ileum with secondary intussusception. A laparoscopic small bowel resection was performed. Histology revealed a large pedunculated polypoidal mass measuring 4 × 2.5 × 2 cm consistent with a submucosal lipoma. She had complete resolution of her symptoms and remained well at 12-month follow-up.

**Conclusion:**

This case highlights an unusual cause of incomplete small bowel obstruction successfully treated through interdisciplinary cooperation. Ileal lipomas are not typically amenable to endoscopic removal and require resection. This can be successfully achieved via a laparoscopic approach with early restoration of premorbid functioning.

## Introduction

Neoplasms of the small intestines are rare [1]. Gastrointestinal lipomas are benign tumors that can occur in the small bowel but occur most commonly in the colon. The majority are asymptomatic and are detected incidentally on abdominal imaging. Removal is warranted if tissue diagnosis is deemed essential or if severe symptomatology, such as pain or bleeding, exists [2].

We report a case of terminal ileal lipoma causing intermittent intussusception in a 52-year-old woman. The lipoma was diagnosed at ileocolonoscopy and successfully removed through laparoscopy. A review of the literature on small bowel intussception and gastrointestinal (GI) lipomas is also presented in this report.

## Case presentation

**Figure 1:**
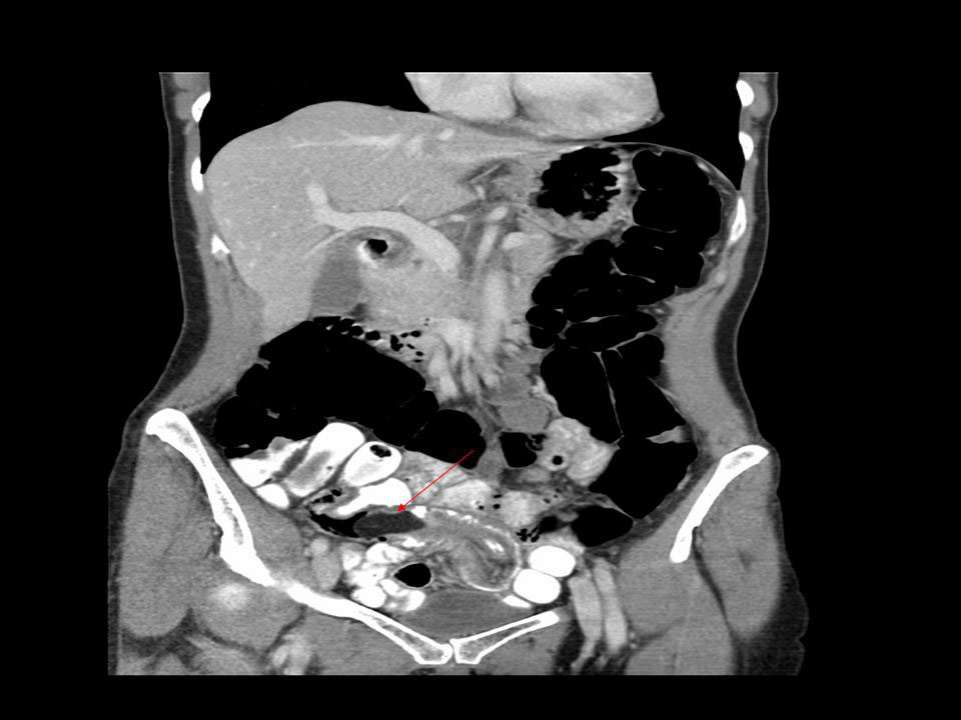
**Contrast-enhanced computed tomography scan of the abdomen demonstrates a smooth-walled, low-density intramural lesion**. It measures 3.2 × 1.6 cm. The ileum at the proximal end of the lesion is mildly dilated with a centrally placed narrowed channel of contrast consistent with an intussusception.

A 52-year-old Irish Caucasian woman presented with a three-month history of intermittent central abdominal pain and constipation. She did not describe gastrointestinal bleeding or weight loss. She previously underwent a transabdominal hysterectomy for menorrhagia. Her physical examination was unremarkable. Initial investigations, such as blood tests, abdomen ultrasound and gastroscopy were unremarkable. Ileocolonoscopy revealed a pedunculated terminal ileal lesion prolapsing into her caecum. Computed tomography (CT) of her abdomen and pelvis demonstrated a smooth-walled, low-density, intramural lesion in the terminal ileum. It measured 3.2 × 1.6 cm. The ileum at the proximal end of the lesion was mildly dilated with a centrally placed narrowed channel of contrast, which was consistent with an intussusception possibly secondary to an intramural lipoma. There was no evidence of obstruction (Figure 1).

**Figure 2:**
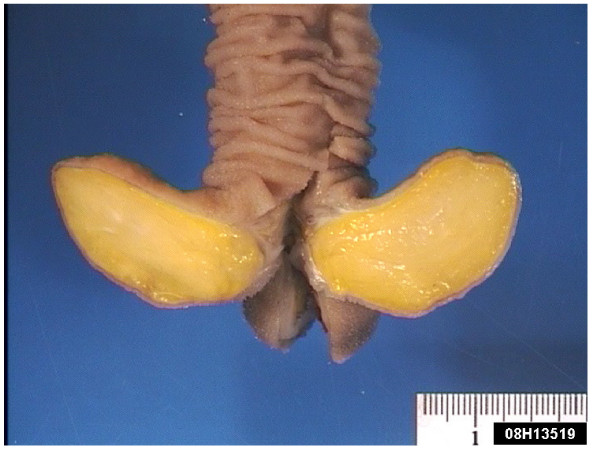
**Macroscopic view of a large pedunculated polypoid mass arising from the luminal surface of the ileal resection specimen**. Appearances are consistent with a lipoma.

She underwent an elective laparoscopic small bowel resection and stapled functional end-to-end anastomoses. On macroscopy the lesion appeared as a large pedunculated polypoid mass measuring 4 × 2.5 × 2 cm with focal mucosal ulceration (Figure 2). Microscopy revealed a submucosal lipoma with blunting of the overlying mucosal villi and pyloric gland metaplasia. She made an uneventful recovery and was discharged home on the fourth postoperative day. She returned to work on the 12^th ^postoperative day. She remained free of symptoms at three-month follow-up.

## Discussion

Lipomas are benign tumors of mesenchymal origin. They are the second most common benign tumors in the small intestine and account for 10% of all benign gastrointestinal tumors and 5% of all gastrointestinal tumors. They are predominantly submucosal and protrude into the lumen [2]. Occasionally, they arise in the serosa. Gastrointestinal lipomas are most commonly located in the colon (65% to 75%, especially on the right side), small bowel (20% to 25%), and occasionally in the foregut (<5%) [2]. Lipomas are largely asymptomatic. Major presenting features are intestinal obstruction and hemorrhage [3].

Intussusception in adults is a rare entity that it is generally caused by definable intraluminal pathology [4]. Diagnosis can be challenging. Intussusception is classified according to its gastrointestinallocation: enteric, ileocaecal, or colonic [4]. In ileocaecal intussusceptions, the ileocaecal valve acts as the lead point. The ileum ('intussusceptum') telescopes into the colon ('intussuscipiens') through the ileocaecal valve [5,6]. Intussusception leads to the development of venous and lymphatic congestion, which results in intestinal edema. If not treated promptly, the arterial blood supply to the bowel will be compromised, thus leading to ischaemia, perforation and peritonitis [4]. Only 5% of all intussusceptions occur in adults [7]. In 90% of these cases a predisposing lesion is identified [7]. This is contrary to intussusception in the pediatric population where an organic lesion is found in only 10% of documented cases [3]. In adults, it is important to differentiate between small bowel and colonic intussusception. In 63% of cases of small bowel intussusceptions, a benign underlying lesion can be found. Meanwhile, a malignant etiology has to be expected in 58% of cases of large bowel intussusceptions [8].

Lipomas can be diagnosed through conventional endoscopy, capsule endoscopy, barium studies and, most importantly, CT. Typical endoscopic features are smooth, yellowish surface with pedunculated or sessile base, as seen in this case. Other endoscopic characteristics are the "cushion sign" and "naked fat sign" [2]. CT usually reveals a smooth, well-demarcated sausage-shaped mass. It may also reveal associated intussusception if present [5]. Capsule endoscopy and digital balloon endoscopy are newer means for diagnosing lipomas and are particularly helpful in cases involving small bowel lipomas [2]. Associated intussusception can be confirmed on contrast enema ('crescent sign'), CT and magnetic resonance imaging (MRI). Multislice CT facilitates the assessment of vascular supply to the affected bowel loop in cases of intussusception where impending ischemia is suspected [4].

The treatment for lipomas depends on the clinical manifestations. Indications for their removal include intestinal obstruction, hemorrhage and malignant potential [4]. There is a theoretical risk of sarcomatous change but this has rarely been documented in the literature [1]. Endoscopic removal is possible but potentially complicated. In view of the submucosal location, there is an inherent risk of perforation [9]. Furthermore, lipomas have high water content, which means a large amount of cautery is necessary to achieve effective hemostasis [9]. Surgery can be performed through laparoscopy or via an open approach. The type of resection and anastomosis depends on the location, bowel wall integrity, and vascular supply of the lipoma [6]. Elective laparoscopic resection of lipomas is the treatment of choice with the concomitant benefits of laparoscopic surgery, such as shorter duration of hospital stay, less postoperative pain, early restoration of (GI) function and good cosmesis [6].

## Conclusion

In this case, we illustrate the importance of a thorough interdisciplinary evaluation of patients with midgut abdominal pain. It highlights the diagnostic values of CT scanning and completed ileocolonoscopy. Despite preoperative localization, laparoscopy facilitates a thorough evaluation of the intraperitoneal contents and therapeutic resection of the affected segment. This report confirms the recognized benefits of laparoscopic surgery with associated early return to premorbid functioning. In patients with persistent episodes of incomplete intestinal obstruction, atypical causes, such as the etiology we describe here, should be considered.

## Consent

Written informed consent was obtained from our patient for publication of this case report and any accompanying images.

## Competing interests

The authors declare that they have no competing interests.

## Authors' contributions

NOA contributed in collecting the requisite literature and wrote the case report. DOK also collected the requisite literature and reviewed the literature. DCM also contributed in collecting the requisite literature. BR and PCN were involved in the diagnosis of our patient. PCN also performed the surgery. All authors read and approved the final manuscript.
